# Can Agricultural Productive Services Promote Agricultural Environmental Efficiency in China?

**DOI:** 10.3390/ijerph19159339

**Published:** 2022-07-30

**Authors:** Yingyu Zhu, Junmiao Deng, Menghan Wang, Yuanchang Tan, Wei Yao, Yan Zhang

**Affiliations:** 1 College of Economics and Management, Shenyang Agricultural University, Shenyang 110866, China; zhuyingyu@stu.syau.edu.cn (Y.Z.); wangmenghan0808@stu.syau.edu.cn (M.W.); tanyuanchang254@stu.syau.edu.cn (Y.T.); yaowei77@stu.syau.edu.cn (W.Y.); 2 School of Economics and Trade, Henan University of Technology, Zhengzhou 450001, China; miaomiao_75@haut.edu.cn; 3 Institute of Higher Education, Shenyang Agricultural University, Shenyang 110866, China

**Keywords:** green agriculture, agricultural productive services, net carbon sinks, agricultural environmental efficiency

## Abstract

Agricultural productive services are important paths to realize the development of green agriculture, while the effect of agricultural productive services on the agricultural environment and its influencing mechanism are not yet clear. With the panel data of agricultural production in China from 2004 to 2019, by using multi-output stochastic frontier analysis with an output-oriented distance function, this study investigates agricultural environmental efficiency based on net carbon sinks. Then, this study explores the effect of agricultural productive services on agricultural environmental efficiency and its mechanisms by adopting ordinary least squares regression with fixed-effect panel model, causal steps approach, and spatial econometric method. The main findings are as follows: Firstly, agricultural productive services enhance agricultural productivity and agricultural environment by optimizing inputs and increasing outputs, and thus improve agricultural environmental efficiency. This result holds steadily after using instrumental variables to deal with endogeneity, changing the measurement of the dependent and independent variables, and subdividing the sample. Secondly, the pathways of agricultural productive services affecting agricultural environmental efficiency are mainly reflected in technology progress, planting structure adjustment, factor allocation optimization, and spatial spillover. Thirdly, due to the law of diminishing marginal returns, the impact of agricultural productive services on agricultural environmental efficiency is more significant when the level of agricultural productive services is relatively low. To improve agricultural environmental efficiency, we suggest implementing different productive service strategies in different regions, strengthening information integration, and improving infrastructure.

## 1. Introduction

Along with the increasing attention paid to environmental issues, the agricultural environment has gradually become one of the most attractive research topics in the agricultural field in China. Agricultural environment efficiency, which brings environmental factors into traditional agricultural technical efficiency, is an effective index to balance agricultural development and agricultural environment. Especially since China has set forth the aims of carbon peak in 2030 and carbon neutrality in 2060, agricultural environmental efficiency based on carbon emissions and its determinants have caused increasing concern. Existing fruitful studies on the determinants of agricultural environmental efficiency are developed from two aspects of the external agricultural environment and the internal productive factors. In terms of the external environment, agricultural development level [1,2], urbanization, natural disasters, production risk [3], innovation [4], and energy price [5] are taken into account. In terms of internal factors, agricultural structure [2], agricultural labor force, reservoir infrastructure construction, agricultural mechanization service organizations [5], as well as mechanization [5,6,7] are frequently mentioned.

At the same time, due to the ubiquity of small-scale and fragmented farming, agricultural productive services adapted to China’s national conditions have spread and popularized rapidly. Agricultural productive services are an essential driving force for improving agricultural productivity and promoting agricultural modernization [8], for its benefits of realizing economies of scale and reducing agricultural production costs without changing the social security functions of cropland [9,10]. Nevertheless, agricultural productive services mainly rely on large machinery that consumes diesel and other fuels, emitting large amounts of carbon dioxide. It is worth noting that the fuel consumption of energy activity has been one of the most fundamental origins of agricultural carbon emissions [11]. However, few studies concentrate on whether the promotion of agricultural productive services exerts a negative impact on the agricultural environment and causes the variation of agricultural environmental efficiency. To date, it is still unclear whether agricultural productive services will lead to the improvement of the agricultural environment. Therefore, more imperative exploration of whether agricultural productive services improve agricultural environmental efficiency from a holistic evaluation perspective can not only conduce to understanding the formation and evolution of agricultural environmental efficiency, but also help to promote the relevant policies for the promotion and application of agricultural productive services.

Agricultural environmental efficiency is determined by agricultural inputs, outputs, and carbon emissions [7], which are influenced by agricultural productive services. In re-ality, agricultural productive services, also known as agricultural production outsourcing, refer to outsourcing some or all agricultural production stages to service providers or other farmers [12]. Due to its incremental, overlapping, complementary advantages [13], agri-cultural productive services can help optimize agricultural factor inputs while keeping agricultural output increased or unchanged. This not only reduces agricultural production costs [14], but also reduces carbon emissions by optimizing agricultural chemical inputs. In addition, more generally, the cost of agricultural productive services to a service provider is lower than the cost of labor to complete the same task because of specialization. Accordingly, through the adoption of productive services, farmers can not only save production costs, but also save labor time to engage in non-agricultural work to obtain more income [15]. Moreover, agricultural production services embed green production techniques and green production materials in farmers’ production processes, change their customs and experience with fertilizer application, and thus achieve a reduction in agricultural chemical materials. In addition, agricultural productive services have a mix of benefits, such as increasing the speed of operations, enhancing the timeliness of crucial production stages, improving the ability to cope with weather-related risks, and reducing losses in the harvest process, which exert positive effects on agricultural output [13]. Machila et al. revealed that agricultural outsourcing had a significant impact on farmers’ crop income and net crop income in Zimbabwe [16]. Agricultural outsourcing contributes significantly and substantially to household crop income and the net income of farmers who participated in the program of outsourced extension service [17]. From the perspective of economics, farmers share land operation rights with service providers by purchasing socialized agricultural services, which benefit from specialization arising from the division of labor [14] and will inevitably affect agricultural production efficiency and agricultural environmental efficiency. Agricultural productivity increased by 25.61% for China’s farmer households who chose agricultural productive services, and the productivity would increase by 10.86% if non-outsourcing farmer households chose to outsource [18].

In summary, the literature on the impact of agricultural productive services on agricultural inputs and outputs are growing. However, the research on agricultural environmental impact is still scarce, which needs further theoretical analysis and empirical verification. In particular, carbon emissions are the inevitable product of modern agricultural production for null-jointness and weak disposability imposed on agricultural production. It means that the impact of agricultural productive services on agricultural environmental efficiency requires not only further theoretical analysis but also more micro-empirical results for verification. To fill this observed deficiency, this paper empirically examines the effects of productive agricultural services on agricultural environmental efficiency. Furthermore, comprehensive theoretical analyses and empirical tests are needed to explain the environmental effects of agricultural productive services, i.e., the mechanisms by which agricultural productive services contribute to agricultural environmental efficiency. To make up for this theoretical gap, this paper presents a comprehensive overview of the possible influencing pathways, and empirically validates some of them.

In these contexts, based on the panel data of 30 provinces in mainland China from 2004 to 2019, this paper investigates the role of agricultural productive services in improving agricultural environmental efficiency. The findings indicate that agricultural productive services significantly improve agricultural environmental efficiency, which holds steady after endogeneity treatment and a series of robust analyses. Meanwhile, the impact of agricultural productive services on agricultural environmental efficiency shows a marginal decreasing trend. Further research conclusions suggest that the spread of agricultural productive services affects both agricultural inputs and outputs through promoting technology progress, changing cropping structure, and optimizing factor input structure. The above pathways have significant spatial spillover effects.

This paper contributes to the existing research from the following three aspects. First, we examine the heterogeneity of agricultural productive services affecting agricultural environmental efficiency based on accurately measuring agricultural environmental efficiency, expanding not only the study of the effects of agricultural productive services, but also the analysis of the factors influencing agricultural environmental efficiency. Second, we develop an analytical framework for the impact of agricultural productive services on agricultural environmental efficiency, namely that agricultural productive services affect agricultural environmental efficiency through three aspects, i.e., inputs, outputs, and environmental factors. Third, we propose the main mechanisms through which agricultural productive services affect agricultural environmental efficiency at a theoretical level and test empirically through the causal steps approach for the mediating effect test.

The remainder of this paper is structured as follows: the “Methodology and Data” section describes analysis framework, empirical methods, and the nature of data. Econometric results and discussion are presented in the “Results” and “Discussion” sections, and the “Conclusions” section sets out the main conclusions and some policy implications.

## 2. Methodology and Data

### 2.1. Analysis Framework of Agricultural Productive Services Affecting Agricultural Environmental Efficiency

In order to reveal the relationship between agricultural productive services and agricultural environmental efficiency in depth, we attempt to explore the influencing pathways of agricultural productive services on agricultural environmental efficiency from four aspects.

Firstly, agricultural environmental efficiency is influenced by agricultural productive services through agricultural technology progress. Most farmers in China face high labor costs and credit constraints, making it challenging to purchase advanced and costly agricultural machinery and choose the reduction scheme for agricultural chemical materials. Agricultural productive services are the best solutions to these problems by reducing the cost of purchasing costly agricultural equipment and lowering the threshold for adopting advanced agricultural technology. On the one hand, the agricultural technology progress, which contains advanced farming technology and advanced energy technology, conduces to improve energy efficiency and directly reduces carbon emissions from energy consumption. On the other hand, agricultural technology progress can indirectly reduce carbon emissions by optimizing the structure of agricultural energy and fertilizer consumption. For example, the growing use of renewable energy can reduce the carbon emission intensity per unit of energy, and the increasing use of new biological fertilizers can increase the carbon sequestration capacity of soil.

Secondly, agricultural environmental efficiency is influenced by agricultural productive services through agricultural planting structure adjustment. Agricultural productive services help to accelerate the large-scale development of agriculture, promote the proportion of grain sown area [19], and achieve the agglomeration of planting varieties. On the one hand, research shows that, compared with horticultural crops and cash crops, grain crops require the least input, including labor and fertilizer [19,20]. Consequently, the increase in the proportion of grain crops sown has led to a decrease in the demand for agricultural chemical materials (i.e., pesticides, agricultural films, and fertilizers) per unit area. On the other hand, grain crops are inclined to be produced by large machinery, resulting in an increased demand for diesel fuel consumption. Furthermore, grain crops generally grow more organic matter (i.e., fruit and straw) than cash crops such as vegetables and flowers, and thus have a more substantial carbon sink effect. Therefore, the bigger the cultivated area of grain crops, the higher agricultural environmental efficiency [20].

Thirdly, agricultural environmental efficiency is influenced by agricultural productive services through factor allocation optimization. Agricultural productive services are accompanied by the spatial flow of mechanical resources and the transmission of beneficial agricultural information, and thus realize the effective allocation of regional resources [12]. The popularization of agricultural productive services is accompanied by a shift of agricultural labor to non-agricultural sectors, so the outflow of agricultural labor will strengthen the scarcity of labor as a primary production factor, and then improve the willingness of farmers to use other capitals as a substitute for labor in agricultural production. The decreasing price of machinery relative to labor has led to a decrease in labor input intensity in farm production and an increase in fertilizers, pesticides, and other agricultural chemicals per unit area. Taking fertilizers for example, the outflow of agricultural labor may reduce the frequency of fertilization, but increase the amount of a single fertilization. In addition, the increased use of machinery requires more fuel consumption and brings more carbon emissions, leading to changes in agricultural environmental efficiency. Agricultural productive services contribute to increased specialization in all segments of the agricultural industry chain. The gains from specialization that arise from the division of labor promote the efficiency of agricultural production and realize economies of scale. At the regional level, because of the formation and expansion of productive service markets, agricultural production factors can fully flow and be exchanged between different agriculture operators with the help of agricultural productive services. In other words, agricultural productive services have realized the change of varying factor combinations. The improvement of resource utilization rate eases the excessive use of agricultural chemical resources [21], and thus leads to the change in carbon emissions.

Fourthly, agricultural environmental efficiency is influenced by agricultural productive services through spatial spillover. From a regional perspective, the higher the level of agricultural productive services in a region (i.e., the higher proportion of persons engaged in productive services and the larger market for agricultural productive services), the greater the empowering effect on agricultural producers in the neighborhood. Relying on technological innovation, technology diffusion, specialized division of labor, collaboration, and the similarity of agricultural production resource endowment conditions in adjacent regions, agricultural machinery, which has apparent diffusion and spillover, forms the agricultural agglomeration effect and enhances the network connection effect in neighboring areas.

**Figure 1:**
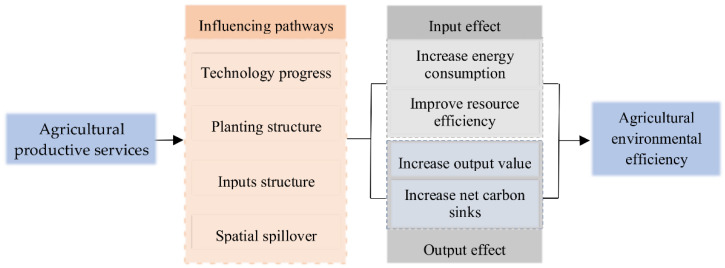
Analysis framework.

Based on the above analysis, we establish an analysis framework (shown in Figure 1) as follows.

### 2.2. Empirical Models

#### 2.2.1. Benchmark Model

To test the relationship between agricultural productive services and agricultural environmental efficiency, the ordinary least squares regression with fixed-effect panel model is employed:

(1) $$AEE_{it}=\alpha_{0}+\alpha_{1}APS_{it}+\sum_{j}\lambda_{j}Z_{itj}+\mu_{i}+\eta_{t}+\xi_{it}$$

where $AEE_{it}$ represents the agricultural environmental efficiency of province *i* at year *t*, $APS_{it}$ denotes the level of agricultural productive services, $Z_{it}$ refers to control variables; $\alpha_{0}$ is the intercept term, $\alpha_{1},\lambda_{j}$ are the estimation coefficients of explanatory variable and control variables, $\mu_{i}$ and $\eta_{t}$ represent the fixed effects of province and year, and $\xi_{it}$ is a stochastic error term.

#### 2.2.2. Causal Steps Approach for Mediating Effect Test

To explore the pathways of agricultural productive services affecting agricultural environmental efficiency, based on the test method of Baron and Kenny (1986) [22], the causal steps approach for mediating effect test is set as follows:

(2) $$M_{it}=\beta_{0}+\beta_{1}APS_{it}+\sum_{j}\lambda_{j}Z_{itj}+\mu_{i}+\eta_{t}+\xi_{it}$$

(3) $$AEE_{it}=\gamma_{0}+\gamma_{1}APS_{it}+\gamma_{2}M_{it}+\sum_{j}\lambda_{j}Z_{itj}+\mu_{i}+\eta_{t}+\xi_{it}$$

where $M_{it}$ represents the mediating variables, $\beta_{0},\gamma_{0}$ are the intercept terms, and $\beta_{1},\gamma_{1},\gamma_{2},\lambda_{j}$ are the estimation coefficients of corresponding variables. Equation (2) is used to test the effect of the independent variable on the mediating variables, and Equation (3) is used to test the effect of the independent variable on the dependent variable after introducing mediating variables. If the regression coefficient on agricultural productive services decreases or becomes insignificant, it indicates that the impact of agricultural productive services on agricultural environmental efficiency comes partly or entirely through the pathway of mediating variable.

#### 2.2.3. Spatial Econometric Model

We employ the Spatial Dubin Model with fixed effects to verify the spatial spillover effect of agricultural productive services on agricultural environmental efficiency:

(4) $$AEE_{it}=\alpha_{0}+\tau AEE_{i,t-1}+\rho WAEE_{it}+\alpha_{1}APS_{it}+\alpha_{2}WAPS_{it}+\sum_{j}\lambda_{j}Z_{itj}+\mu_{i}+\eta_{t}+\xi_{it}$$

where *W* represents the spatial weight matrix, and geographical distance matrix is adopted in the paper. τ is the first-order lag coefficient of dependent variable, ρ is the spatial correlation coefficient, and $\alpha_{1},\alpha_{2},\lambda_{j}$ are the estimated coefficients of each explanatory variable.

#### 2.2.4. Endogeneity and Two-Stage Least Squares

Biases might remain in empirical models because of two types of endogenous problems. The first is the omitted variable problem, i.e., some variables that affect both agricultural productive services and agricultural environmental efficiency are omitted from the regression model. The second is reverse causality, i.e., agricultural environmental efficiency might affect agricultural productive services. For example, provinces with high agricultural environmental efficiency level are usually regions with the high level of factor endowments. These regions, due to the high level of regional development and high price relative to labor, will adapt to local agricultural development requirements through large-scale adoption of agricultural productive services, which in turn contribute to the improvement of their agricultural productive services. This means that the core independent variable and the dependent variable suffer from reverse causality [23].

To solve the problem of possible omitted variables, we employ a fixed-effect model with panel data to control the unobservable effects at provincial and time level, while adding as many control variables as possible in the regression analysis based on existing studies, such as rural human capital and production risk. To solve the problem of reverse causality, we introduce a suitable instrumental variable and employ two-stage least squares method to alleviate endogenous problems. The first stage is used to obtain prediction to replace endogenous variables, and the second stage is used to draw the final estimation result.

### 2.3. Variables

#### 2.3.1. Dependent Variable: Agricultural Environmental Efficiency

Following Zhu et al. [7], we adopt the multi-output stochastic frontier analysis method, which is based on the output-oriented distance function, to obtain agricultural environmental efficiency. In terms of the production function form, we introduce a time-varying parameter model to estimate elastic changes across time accurately. Drawing on the traditional literature [24,25], input variables and output variables are selected to calculate agricultural environmental efficiency. The main input variables of agricultural production are labor, machinery, fertilizer, land and fuel, which are measured by the number of employees (in millions), the total power of planting machinery (in million kilowatts), the sum of the gross weight of various fertilizers (in million tons), the sown area (in million hectares) reflecting the actual utilization of the cultivated land, and diesel oil (in million tons) in the planting industry, respectively. We employ the gross value of output (in million CNY) and net carbon sinks (in million tons of CO_2_-equivalent) in the planting industry as the output variables because agriculture has the attribute of net carbon sinks. The calculation formula, coefficient of agricultural carbon sinks, and carbon emissions are based on the research of Zhu et al. (2022) [26].

**Figure 2:**
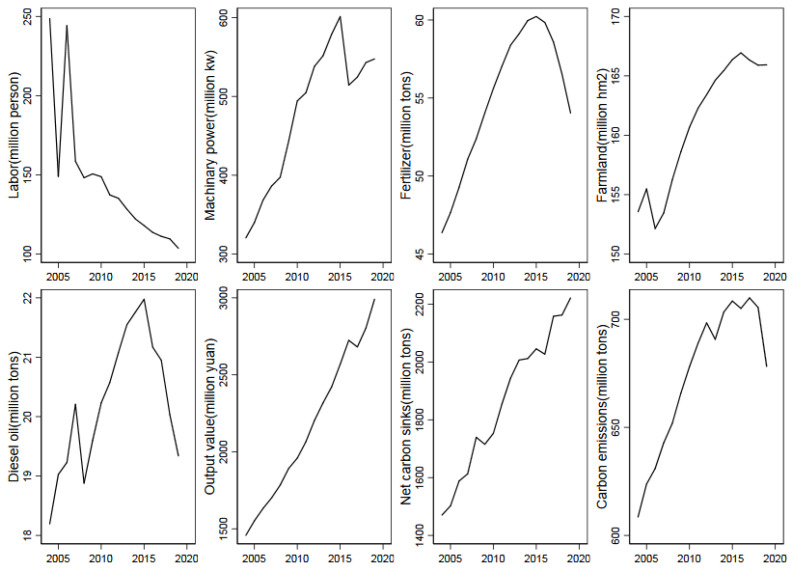
The changing trend of agricultural inputs and outputs in China from 2004 to 2019.

The descriptive statistics on agricultural input and output variables on provincial level are shown in Table 1, and the changing trends of agricultural inputs and outputs on national level in China from 2004 to 2019 are shown in Figure 2.

#### 2.3.2. Independent Variable: Agricultural Productive Services

From the point of view of the demand side (i.e., the objects of agricultural productive services), the sown area and the number of farmers adopting productive services are the ideal indexes to measure the level of productive services; from the point of view of the supply side, the supplier (i.e., the number of agricultural machinery service organizations and agricultural machinery professional service households or organizations) and the power of agricultural machinery for productive services are reasonable indicators. Limited by data availability, we employ the number of people engaged in agricultural productive services per unit area to measure the level of agricultural productive services.

#### 2.3.3. Instrumental Variable

To solve the problem of endogeneity, the usual approach is to introduce a suitable instrumental variable [23]. Following Zhu et al. [7], this paper adopts the ratio of road mileage to farmland area in the region as an instrumental variable for agricultural productive services. As the transportation infrastructure, roads do not directly impact agri-cultural output value and net carbon sinks, but they can improve the degree of agricultural productive services via improving road conditions and reducing transportation costs for agricultural machinery.

#### 2.3.4. Mediating Variables

Based on the analysis in the “Literature Review” section, technology progress, planting structure, and input structure are selected to study the influencing mechanism of agricultural productive services affecting agricultural environmental efficiency. Agricultural machinery, which contains advanced planting and harvesting techniques, is accompanied by the transfer and diffusion of management techniques in its service process. Consequently, technology progress is characterized by the ratio of the total power of machinery to farmland area. There are significant differences between cash crops and grain crops in mechanical demand and agricultural chemical materials input [5], so the planting structure is represented by the ratio of grain sowing area to crop sowing area. The main agricultural inputs include machinery, farmland, labor, and chemical fertilizer. Considering the relevance of machinery and the stability of farmland, the ratio of fertilizer to labor per unit area is used to characterize the input structure.

#### 2.3.5. Control Variables

Agricultural operation scale, planting industry development level, rural human capital, production risk, regional economic development level, part-time employment of labor, and urban-rural income gap are controlled in our models. Agricultural operation scale (in hectare per household), which is an essential factor affecting the consumption of farm machinery and chemical materials [11], is represented by the ratio of farmland to rural households, and its square term is introduced to examine the possible threshold of land. Based on the viewpoint of comparative advantage, the development level of the planting industry is measured by the share of the planting industry in agriculture. As a factor with strong positive externalities, rural human capital (in years) is calculated as the average length of schooling of the rural population. As farmers will increase fertilizer and pesticide inputs to avoid risks [3,27], production risk is selected as the control variable, which refers to the ratio of the affected area to the total sown area of crops. Regional economic development level (in CNY per person), which affects carbon emissions intensity [1], is expressed by the ratio of a region’s GDP to its population. As an essential factor affecting the capital inputs (i.e., machine, chemical fertilizer, and pesticides) in agricultural production [11], the part-time employment of labor is measured as the proportion of wage income of rural residents. The urban-rural income gap, which leads to the flow of the labor to cities [28], is represented by the ratio of disposable income of urban residents to rural residents. In addition, due to the complexity of agricultural production, agricultural environmental efficiency will be affected by many unobserved factors, such as social culture and natural conditions [29], so provincial and year fixed effects are controlled too.

### 2.4. Data

From the time perspective, agricultural mechanization and agricultural productive services have developed rapidly in China since 2004, and the data of agricultural productive services have been included in the national statistical yearbook since then. Therefore, the data employed in this study are the province-by-year panel of 30 provinces of mainland China from 2004 to 2019, and Tibet, Hong Kong, Macao, and Taiwan are excluded due to data unavailability. The annual data for each province associated with agricultural production are obtained from the annual *China Rural Statistical Yearbook* and *China Agricultural Machinery Industry Yearbook*. In addition, price variables are deflated according to the price level in 2004.

**Figure 3:**
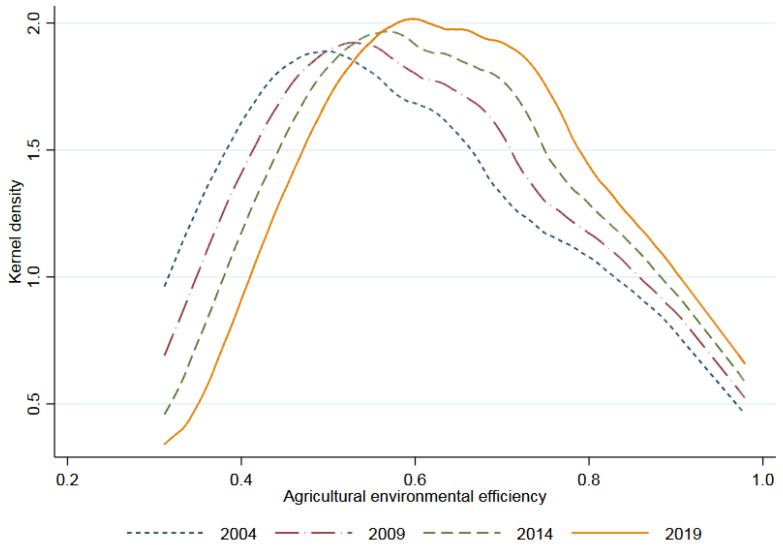
Kernel density of agricultural environmental efficiency.

**Figure 4:**
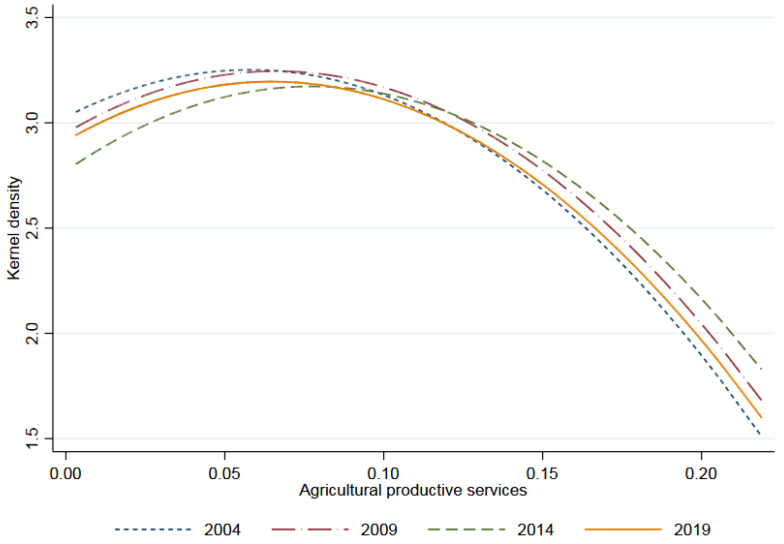
Kernel density of agricultural productive services.

The descriptive statistics on variables are shown in Table 2. The differences of agricultural environmental efficiency and agricultural productive services among different provinces are presented in Figure 3 and Figure 4, showing that there are great differences between samples, which are suitable for regression analysis.

## 3. Results

### 3.1. Benchmark Regression Results

The regression results in columns (1) and (2) of Table 3 present the parameter estimates for the benchmark regression based on Equation (1). Among them, column (1) does not include control variables, while column (2) adds control variables, with both columns controlling province and time differences. As shown in column (1), the coefficient on agricultural productive services is positive and statistically significant at the 1% level, indicating that the development of agricultural productive services promotes the level of agricultural environmental efficiency. However, the analysis is likely to be confounded by omitted variables. After introducing control variables, the results in column (2) show that the coefficient on agricultural productive services is still positive and statistically significant at the 1% level. To be explicit, the augmentation of agricultural productive services by 1% leads to an increase of 7.16% in agricultural environmental efficiency. This means that, despite the increasing effect in carbon emissions caused by energy consumption, the increasing carbon sink effect, which is brought about by the increase of output and the reduction of agricultural chemical inputs, is more potent. In fact, the carbon emissions of China’s planting industry began to decline after 2016, but the carbon sinks have been increasing, showing an obvious net carbon sink effect.

Table 3 also identifies some statistically significant control variables. An inverted U-shaped relationship exists between farmland operation scale and agricultural environmental efficiency. With the expansion of farmland operation scale, agricultural environmental efficiency gradually improves, which is unsurprising as the appropriate expansion of farmland operation scale is conducive to the rational allocation of production factors by agricultural producers and the reduction of material waste. When farmland operation scale exceeds the threshold value, agricultural environmental efficiency declines. The reason lies in the difficulty of managing and realizing the maximum potential if the farmland per capita is too large [30]. There is a significant positive relationship between rural human capital and agricultural environmental efficiency at a 1% level. This is due to the considerable positive externality of human capital, which has a significant incremental improvement on environmental quality. Production risk is significantly and negatively related to agricultural environmental efficiency. The greater the frequency and degree of natural disasters, the greater the production risk perceived by farmers, and the more likely it is to use agricultural chemical materials to avoid risks and reduce losses, resulting in the decline of environmental efficiency. The high degree of part-time labor employment and the low degree of urban-rural income gap improve agricultural environmental efficiency through higher demand for productive services.

### 3.2. Two-Stage Least Squares Results

Columns (3) and (4) of Table 3 present the parameter estimates for the two-stage least squares method. The results of the first stage show a significant positive correlation between the instrumental variable and agricultural productive services, whether with or without control variables, which is consistent with the hypothesis of correlation of instrumental variables. The results of the second stage regression showed that agricultural productive services still had a significant contribution to agricultural environmental efficiency, which confirmed the robustness of the benchmark regression results.

### 3.3. Robust Analysis Results

To further verify the robustness of the impact of productive agricultural services on agricultural environmental efficiency, we conduct the robustness analysis from three aspects: replacing the dependent variable, replacing the core independent variables, and subdividing the sample into main and non-main grain-producing areas.

Firstly, we recalculate agricultural environmental efficiency using the translog production function and re-estimate the impact of agricultural productive services. The results in column (1) of Table 4 presents that the effect of agricultural productive services on agricultural environmental efficiency remains positive and significant at a 1% level.

Secondly, we adopt the number of productive service institutions per unit area to express the level of agricultural productive services. The results in column (2) of Table 4 show that the independent variable has a positive correlation with agricultural environmental efficiency with significance at a 5% level, which further confirms the contribution of agricultural productive services to agricultural environmental efficiency.

Thirdly, we divide the samples into main and non-main grain-producing areas. There are 13 provinces in main grain-producing areas, such as Heilongjiang and Henan, with a high level of agricultural productive services. The coefficients on agricultural productive services are positive and statistically significant at least at a 5% level in columns (3) and (4) of Table 4, but differ a lot in the main grain-producing areas and non-main grain-producing areas. The coefficient on agricultural productive services is 0.1394 in non-main grain-producing areas, which is more significant than that of main grain-producing areas. This means that the marginal effect of agricultural productive services is somewhat larger in non-main grain-producing areas, i.e., agricultural productive services in non-main grain-producing areas are still in the early development stage and have ample space for growth.

### 3.4. Mechanism Analysis Results

To examine the transmission mechanism of agricultural productive services to agricultural environmental efficiency, we employ Equations (2) and (3) for mediating effect tests from three perspectives of technology progress, planting structure, and input structure, and adopt the spatial econometric model for the spatial spillover effect test.

Table 5 reports the results of the mechanism analysis. The regression results of the first step are presented in columns (1), (2), and (3). The coefficients are positive and significant at a 1% level, indicating that the increase in agricultural productive services has significantly improved technology progress, and optimized the structure of planting crops and factor inputs. Column (4) exhibits the impact of agricultural productive services on agricultural environmental efficiency without mediating variables, and columns (5), (6), and (7) demonstrate the regression results of the second step. As shown in column (5), after the inclusion of technology progress, the coefficient on agricultural productive services and its significance become lower, and the coefficient of technology progress is significant at a 5% level, indicating that the mediating mechanism of technology progress is verified. This is because technology progress is a fundamental driver of agricultural productivity and is a critical factor in reducing carbon emissions in agriculture [3,4]. Similarly, the mediating pathways of planting structure and input structure are manifested in columns (6) and (7). Grain crops have a more substantial carbon sink effect and their demand for chemical materials is less than cash crops. Hence, the increase in the share of grain crops sown area has a positive effect on agricultural environmental efficiency. The optimization of inputs structure can effectively reduce the cost of agricultural production and contribute to the improvement of agricultural environmental efficiency.

Table 6 reports the results of Equation (4) under the geographical distance matrix. The spatial coefficient and spatial spillover effects are significantly positive, showing that agricultural productive services have a significant spatial spillover effect on agricultural environmental efficiency, i.e., agricultural productive services in neighboring regions have a positive effect on agricultural environmental efficiency in this region. This reflects that agricultural productive services not only serve the region where they are located, but also radiate to the surrounding regions based on the similar crop structure and the different crop maturity time. Therefore, agricultural productive services not only contribute to local agricultural environmental efficiency, but also promote the efficiency of surrounding regions due to the spatial spillover effect caused by their cross-regional operation.

## 4. Discussion

### 4.1. The Relationship between Agricultural Productive Services and Agricultural Environmental Efficiency

Under the context of sustainable development, it is valuable to investigate agricultural productivity in combination with environmental factors and analyze its critical influencing factors. Our empirical results show that agricultural productive services have a positive impact on agricultural environmental efficiency. Although the existing literature does not directly explore the relationship between them, there are two related types of literature for comparison, both of which have the common basis—mechanization. One is about agricultural mechanization and agricultural environmental efficiency, but the conclusions of their relationship are not consistent. Jiang et al. believed that mechanization led to the decline of agricultural environmental performance based on the provincial data from 2000 to 2017 in China [5]. In the study of Zhu et al., the argument that mechanization and agricultural environmental efficiency presented an inverted U-shaped relationship was verified based on the study sample of China’s 30 provinces during 2001–2019 [7]. The reason for this inconsistency may lie in the inconsistency of the measurement methods and indicators of agricultural environmental efficiency adopted by them. Jiang et al. employed the DEA method and selected carbon emissions and solid residues as environmental outputs [5], while Zhu et al. adopted the SFA method and selected net carbon sinks as environmental outputs [7]. The other is about agricultural green production technologies and agricultural environmental efficiency. The majority of green production technologies in China rely on machinery and energy, which can cause excessive carbon emissions. In the research of He et al., the impact of different green production technologies on low-carbon efficiency is not uniform, and the results vary across regions [31]. For example, mechanized straw returning improves the low-carbon efficiency in the north, while mechanized deep ploughing and scarification improve low-carbon efficiency in the south.

Agricultural environmental efficiency is determined by agricultural productivity and agricultural carbon emissions [31]. To be specific, agricultural inputs and outputs determine agricultural productivity, while agricultural carbon emissions affect agricultural environment [7]. Agricultural productive services, which mainly rely on large machinery that consumes diesel and other fuels, not only affect agricultural performance through inputs and outputs, but also emit large amounts of carbon dioxide from energy consumption. From the perspective of input, as a capital input replacing labor, productive services significantly reduce labor costs and optimize agricultural chemical materials, but increase diesel consumption. From the standpoint of output, most studies confirm the positive effect of productive services on agricultural output [9,19]. From the perspective of carbon emissions, agricultural chemical materials and energy consumption are the two primary sources of carbon emissions in agriculture. The optimization of the former leads to reducing carbon emissions, but the rising diesel consumption results in an increase in carbon emissions. Moreover, the increase effect of carbon emissions is greater than the reduction effect.

The path of agricultural productive services impacting agricultural environmental efficiency is determined by two aspects. The first aspect is to increase agricultural productivity. Studies have confirmed that agricultural productive services are applied to maximize total yield and benefit potential production. At the macro level, Sheng and Chancellor found that the hiring of capital services may lift the productivity of small farms in the Australian grains industry [32]. At the peasant household level, Bravo-Ureta et al. analyzed the impact of a canal irrigation project for smallholders in the Philippines and found that irrigation technology promoted beneficiaries’ production potential [33]. Taking drought-tolerant maize as a research object in the Northern Region of Ghana, Martey et al. revealed that the climate-smart agricultural technologies positively impacted yield and commercialization intensity [34]. The second aspect is to minimize the damage to the agricultural environment. For the planting industry, its attribute of net carbon sink dictates that we should maximize the net carbon sinks rather than minimize carbon emissions.

From the perspective of carbon emissions, the adoption of agricultural productive services can reduce carbon emissions by lowering chemical inputs, but agricultural machinery and its fuel consumption, which support agricultural productive services, directly and indirectly cause carbon emissions. Moreover, in terms of outcome, the carbon emission effect of energy is greater than that of chemical input reduction. However, from the perspective of net carbon sinks, the planting industry showed a prominent positive net carbon sink characteristics and gradually increased.

### 4.2. The Mechanism of Agricultural Productive Services Affecting Agricultural Environmental Efficiency

Technology progress is an essential driving force for productivity growth, and the production frontier will continue to expand outward with technology progress. Taking paddy smallholders in the Philippines, for example, the frontier output was significantly promoted by improved irrigation technology [33]. If there is no change in the productivity of the areas below the production frontier, the environmental efficiency values in these areas will become lower. However, our empirical results show that the environmental efficiency values are improved in almost all regions, suggesting that technology progress plays a role in all regions. Furthermore, the efficiency of the regions with low environmental efficiency rises faster than that of the regions with high environmental efficiency, suggesting that agricultural productive services embodying technology progress play a more significant role in the regions with low environmental efficiency.

Agricultural productive services help to achieve the concentration of crop varieties, especially the increase in the proportion of the sown area of grain crops. In general, compared to cash crops, grain crops have higher productivity, relatively fewer chemical inputs, and relatively lower environmental impact. In particular, it is worth mentioning that the carbon absorption rate of grain crops is relatively high, which can easily form more carbon sinks. For example, the carbon absorption rates of wheat and maize are 0.485 and 0.471, respectively, which are higher than that of cash crops (the carbon absorption rate is 0.45).

As a product and cost of agricultural production are dual problems, the optimization of inputs structure means that the cost can be minimized with constant output. We should seek measures to improve agricultural productivity from a cost perspective in the case that the grain output per unit area cannot be significantly increased. Hence, the improvement of agricultural productivity lies in optimizing cost, which depends on the optimal allocation of different input factors. The spread of agricultural productive services has effectively solved the limitation of labor scarcity and realized the rational allocation of resources through the gains from specialization that arise from the division of labor.

On a regional scale, the neighboring regions often have similar crop varieties, and the difference in the distribution along the latitude makes the crop maturity have time differences, which provides a broad market space for agricultural productive services. Agricultural productive services have obvious diffusion and spillover, for they not only serve the local agricultural production, but also provide support for neighboring regions. From the viewpoint of micro-entities, farmers can learn advanced knowledge, technology, and experience from other farmers, agricultural cooperatives, and agricultural production service organizations in adopting agricultural productive services, and thus promote productivity improvement through positive externalities of learning. Employing panel data of 13 prefecture-level cities in Jiangsu province of China from 2000 to 2016, Wu et al. revealed that the spatial spillover effect of mechanization was significant on grain yield due to the cross-regional operation of mechanization [35].

There are several reasons for this paper does not explore the pathway of scale operation, which is analyzed in some other articles [7]. First, the effect of scale operation is not the expansion of land operation scale, but the optimal combination of land and other endowment inputs of farmers. Scale operation at farmer’s level is ultimately reflected in factors input structure, which has been analyzed above. Second, scale operation at the region’s level is reflected in the change of planting structure. The more similar the adjustment of planting structure, the easier it to form a scale agglomeration effect in the region. Third, this paper considers the influence of land operation area among the control variables.

To the best of our knowledge, this is the first attempt to reveal the effect of agricultural productive services on agricultural environmental efficiency and its influencing mechanism at the provincial level in China.

### 4.3. Heterogeneity Impact of Different Levels of Productive Agricultural Services

By comparing the effects of agricultural productive services on agricultural environmental efficiency in different regions in Table 3, we find that the marginal effect of agricultural productive services is more prominent in non-main grain-producing areas. Then, is this because the level of agricultural productive services is lower in non-main grain-producing areas? As a factor of production, do agricultural productive services satisfy the law of diminishing marginal returns? To test these assumptions, we further divide the level of agricultural productive services into three groups, conduct an econometric test (the results are shown in Table 7), and find that the above inferences are valid. This means that, as an input factor, the input of agricultural productive services also conforms to the law of diminishing marginal returns, which is a fundamental law of short-term production. There is an optimal combination of productive services and other input factors. When the ratio is exceeded, the marginal benefit of increasing productive services decreases.

## 5. Conclusions

Based on the panel data of 30 provinces in China from 2004 to 2019, we adopt a multi-output stochastic frontier analysis method to measure agricultural environmental efficiency based on net carbon sink, then explore the effects and mechanisms of agricultural productive services on agricultural environmental efficiency using OLS with fixed-effect panel data, two-stage least squares with instrumental variable, causal steps approach, and a spatial econometric method. The main conclusions are as follows:

Firstly, agricultural productive services have a significant contribution to agricultural environmental efficiency. Agricultural productive services enhance agricultural productivity as well as the agricultural environment through inputs and outputs, and then improve the efficiency of the agricultural environment. This result still holds after using instrumental variables to deal with endogeneity, changing the measurement of dependent and independent variables, and subdividing the sample. Secondly, the mechanism pathways of agricultural productive services affecting agricultural environmental efficiency are mainly reflected in technology progress, planting structure adjustment, factor allocation optimization, and spatial spillover. Advanced agricultural technology and management measures can improve agricultural productivity and energy efficiency, and optimize the energy consumption structure. A reasonable planting structure is conducive to reducing agricultural chemical inputs, improving factor utilization efficiency, and realizing economies of scale. The rational allocation of factors is the fundamental guarantee for the role of agricultural input resources such as labor, farmland, and chemical fertilizer. Agricultural agglomeration and different crop maturity periods make cross-regional services of agricultural productive services possible, thus generating spatial spillover effects. Thirdly, the impact of agricultural productive services on agricultural environmental efficiency is more significant in the regions with low agricultural productive services level because agricultural productive services, as a factor input in agricultural production, conform to the law of diminishing marginal returns.

Given the above evidence and arguments, we draw some policy implications.

Firstly, policy attention should be paid to improving the level of agricultural productive services continuously according to the actual situation in different regions. For regions with low level of agricultural producer services due to terrain factors or crop structure factors, the marginal contribution of agricultural producer services is more pronounced. It is valuable to promote appropriate agricultural productive services in these areas to promote the quality of agricultural development and environmental efficiency. For regions with a high agricultural producer services level, such as main grain-producing areas, the marginal contribution of agricultural producer services is relatively small. The critical measure is to update or replace the existing productive service equipment, and promote the use of agricultural productive services with more advanced technology and higher efficiency. Secondly, efforts should be made to strengthen information diffusion and regional cooperation to realize the positive spatial spillover effect of agricultural productive services. For regions with similar resource endowments and similar crop types, information cooperation is used to guide and promote the cross-regional operation of agricultural productive services, to achieve rational flow and scientific allocation of agricultural machinery. Thirdly, it is necessary to improve the operating conditions of agricultural productive services, such as appropriately increasing the investment in transportation infrastructure, guiding the centralized farmland transfer, and realizing regional agglomeration of crop planting. Improving transportation infrastructure can provide primary conditions and convenience for agricultural machinery operations. Farmland transfer and crop agglomeration are conducive to realizing economies of land scale and service scale in agricultural production. The scale of expansion is beneficial to cultivating the agricultural productive services market and introducing small farmers to modern agriculture.

There are also some potential limitations in this paper. First, in terms of the research scope, this study was conducted at the provincial level. Future research can be conducted at the county level, which potentially has richer data and more accurate results. Second, in terms of the research depth, as agricultural productive services exist in all aspects of agricultural production, such as tillage, sowing, irrigation, fertilization, and harvesting, a possible research direction is to profoundly investigate the impact of different stages of productive services on agricultural environmental efficiency.

## Figures and Tables

**Table 1:** Descriptive statistics on agricultural input and output variables.

Variable Type	Variable	Mean	Std. Dev.	Min	Max
Input variables	Labor (million)	5.062	3.717	0.154	17.555
	Machinary (million kilowatt)	16.232	15.454	0.446	70.656
	Fertilizer (million ton)	1.832	1.416	0.062	7.161
	Farmland (million hectare)	5.363	3.664	0.089	14.903
	Diessel oil (million ton)	0.673	0.672	0.018	4.870
Output variables	Output value (million CNY)	71,574.930	51,567.310	2791.214	242,921.200
	Net carbon sinks(million ton of CO_2_-equivalent)	62.002	56.596	0.789	250.701

**Table 2:** Descriptive statistics on variables.

Variable Type	Variable	Mean	Std. Dev.	Min	Max
Dependent variable	Agricultural environmental efficiency	0.626	0.168	0.311	0.979
Independent variable	Agricultural productive services	0.069	0.048	0.003	0.219
Mediating variables	Technology progress	0.400	0.196	0.048	1.077
	Planting structure	0.652	0.131	0.328	0.971
	Inputs structure	0.406	0.172	0.073	1.173
Control variables	Agricultural operation scale	6.849	6.109	0.929	30.643
	Planting industry development level	0.567	0.090	0.339	0.772
	Rural human capital	7.480	0.687	5.144	9.731
	Production risk	0.208	0.148	0.000	0.936
	Regional economic development level	0.011	0.006	0.003	0.033
	Part-time employment of labor	18.138	22.073	0.062	76.300
	Urban-rural income gap	2.857	0.563	1.850	5.120

**Table 3:** Impact of agricultural productive services on agricultural environmental efficiency.

Variables	OLS		2SLS	
	(1)	(2)	(3)	(4)
Agricultural productive services	0.332 ***	0.072 ***	1.768 ***	0.962 ***
	(0.055)	(0.026)	(0.176)	(0.180)
Agricultural operation scale		0.002 **		0.008 ***
		(0.001)		(0.002)
Square of agricultural operation scale		−0.000		−0.000 **
		(0.000)		(0.000)
Planting industry development level		−0.002		−0.052
		(0.019)		(0.037)
Rural human capital		0.021 ***		0.017 ***
		(0.002)		(0.003)
Production risk		−0.010 **		−0.007
		(0.005)		(0.009)
Regional economic development level		0.687		−2.949 **
		(0.490)		(1.165)
Part-time employment of labor		0.000 ***		0.000 ***
		(0.000)		(0.000)
Urban-rural income gap		−0.032 ***		−0.029 ***
		(0.002)		(0.005)
Constant	0.603 ***	0.531 ***		
	(0.004)	(0.020)		
Fixed province	Yes	Yes	Yes	Yes
Fixed year	Yes	Yes	Yes	Yes
R-squared	0.893	0.841	−1.334	0.419
Coefficient of IV in the first stage			0.028 ***	0.027 ***
			(0.002)	(0.005)
Value F in the first stage			145.200	35.820
Observations	480	480	480	480
Provinces	30	30	30	30

Note: **, ***: statistically significant at 5% and 1%, respectively; Standard error in parentheses.

**Table 4:** Results of robust analysis.

Variables	Changing Dependent Variable	Changing Independent Variable	Non-Main Grain-Producing Areas	Main Grain-Producing Areas
	(1)	(2)	(3)	(4)
Agricultural productive services	0.095 ***	0.107 **	0.139 ***	0.074 **
	(0.027)	(0.045)	(0.047)	(0.031)
R-squared	0.853	0.840	0.837	0.898
Control variables	Controlled	Controlled	Controlled	Controlled
Fixed province	Yes	Yes	Yes	Yes
Fixed year	Yes	Yes	Yes	Yes
Observations	480	480	272	208
Provinces	30	30	17	13

Note: **, ***: statistically significant at 5% and 1%, respectively; Standard error in parentheses.

**Table 5:** Results of the causal steps approach for mediating effect test.

Variables	Technology Progress	Planting Structure	Inputs Structure	Agricultural Environmental Efficiency			
	(1)	(2)	(3)	(4)	(5)	(6)	(7)
Agricultural productive services	1.346 ***	0.269 ***	0.768 ***	0.072 ***	0.046 *	0.060 **	0.037
	(0.165)	(0.088)	(0.133)	(0.026)	(0.028)	(0.026)	(0.026)
Technology progress					0.019 **		
					(0.008)		
Planting structure						0.043 ***	
						(0.014)	
Inputs structure							0.045 ***
							(0.009)
R-squared	0.620	0.201	0.486	0.841	0.843	0.844	0.849
Control variable	Controlled	Controlled	Controlled	Controlled	Controlled	Controlled	Controlled
Fixed province	Yes	Yes	Yes	Yes	Yes	Yes	Yes
Fixed year	Yes	Yes	Yes	Yes	Yes	Yes	Yes
Observations	480	480	480	480	480	480	480
Provinces	30	30	30	30	30	30	30

Note: *, **, ***: statistically significant at 10%, 5% and 1%, respectively; Standard error in parentheses.

**Table 6:** Results of the spatial econometric model.

Spatial Coefficient	LR_Direct	LR_Indirect	LR_Total	R-Squared
0.673 ***	0.048 **	0.569 **	0.617 **	0.877
(0.059)	(0.021)	(0.259)	(0.267)	

Note: **, ***: statistically significant at 5% and 1%, respectively; Standard error in parentheses.

**Table 7:** Heterogeneity impact of different levels of productive agricultural services.

Variables	Low Level	Middle Level	High Level
	(1)	(2)	(3)
Agricultural productive services	0.556 ***	0.127	−0.015
	(0.149)	(0.087)	(0.031)
R-squared	0.841	0.871	0.902
Control variable	Controlled	Controlled	Controlled
Fixed province	Yes	Yes	Yes
Fixed year	Yes	Yes	Yes
Observations	160	159	161
Provinces	15	19	15

Note: ***: statistically significant at 1%; Standard error in parentheses.

## Data Availability

The datasets analyzed during the current study are available from the corresponding author on reasonable request.
